# Effects of *Bifidobacterium longum* 35624 in Children and Adolescents with Irritable Bowel Syndrome

**DOI:** 10.3390/nu16121967

**Published:** 2024-06-20

**Authors:** Sylvia Cruchet Muñoz, Sandra Verbeke Palma, Lydia Lera Marqués, María Nelly Espinosa Pizarro, Jacqueline Malig Mechasqui, Katy Sorensen

**Affiliations:** 1 Instituto de Nutrición y Tecnología de los Alimentos “Dr. Fernando Monckeberg”, Área Nutrición Humana, Universidad de Chile, El Líbano 5524, Macul, Santiago 7830490, Chile; scruchet@gmail.com; 2 Escuela de Tecnología Médica, Facultad de Salud, Universidad Santo Tomás, Campus Santiago, Chile. Av. Ejército 146, Santiago 8370003, Chile; 3 Faculty of Graduate Business and Education Programs, Keiser University eCampus, 1900 West Commercial Boulevard. Ste 100, Ft. Lauderdale, FL 33309, USA; lleramarques@gmail.com; 4 Facultad de Medicina, Universidad de los Andes, Monseñor Álvaro del Portillo 12455, Las Condes, Santiago 7620001, Chile; nespinosap@yahoo.es; 5 Hospital Militar de Santiago, Av. Fernando Castillo Velasco 9100, La Reina, Santiago 7880047, Chile; 6 Clínica Red Salud Vitacura de Santiago, Tabancura 1141, Vitacura, Santiago 7650018, Chile; jacquelinemalig@gmail.com; 7 Medical Affairs, Novozymes A/S, Krogshøjvej 36, 2880 Bagsvaerd, Denmark; kass@novozymes.com

**Keywords:** bifidobacteria, disorders of gut–brain interaction, irritable bowel syndrome, probiotics, pediatrics

## Abstract

Irritable bowel syndrome (IBS) and vitamin D deficiency are common among children in Latin America. Previous studies show that *Bifidobacterium longum* **35624**^TM^ improves IBS symptoms in adults. This real-world, single-arm, open-label study conducted in Chile investigated the effects of *B. longum* 35624 (1 × 10^9^ colony-forming units, 12 weeks) on gastrointestinal symptoms (adapted IBS severity scoring system [IBS-SSS]; adapted Questionnaire on Pediatric Gastrointestinal Symptoms [QPGS], and Bristol Stool Form Scale) in 64 children and adolescents (8–18 years) and explored the relationship with baseline vitamin D status. Improvements in all IBS-SSS domains and composite score were observed at week 6 and 12 (*p* < 0.0007 versus baseline), with 98.3% of participants experiencing numerical improvements in ≥3 domains. Clinically meaningful improvement was seen in 96.6% of participants. The distribution of IBS-SSS severity categories shifted from moderate/severe at baseline to mild/remission (*p* < 0.0001). Improvements were not maintained during the two-week washout. Low baseline serum vitamin D levels did not correlate to IBS severity or probiotic response. QPGS significantly decreased from baseline to week 6 (*p* = 0.0005) and 12 (*p* = 0.02). *B. longum* 35624 may improve IBS symptoms in children and adolescents, even those with vitamin D deficiency. A confirmatory randomized controlled trial and further exploration of probiotic response and vitamin D status are needed.

## 1. Introduction

Disorders of gut–brain interaction (DGBI), formerly referred to as functional gastrointestinal disorders (FGIDs), are characterized by recurrent gastrointestinal (GI) symptoms unrelated to organic disease [1]. Often presenting concurrently in childhood [2], DGBI may affect 22% of children worldwide [3] and 12–48% in Latin America [4]. Irritable bowel syndrome (IBS) is among the most common DGBI in children over four years of age [3].

IBS is characterized by recurrent abdominal pain associated with defecation and/or changes in stool form or frequency [5]. Its etiology is complex, involving gut dysmotility, visceral hypersensitivity, altered mucosal and immune responses, central nervous system processing, and gut microbiota composition [1]. Mucosal inflammation triggered by infectious gastroenteritis may induce the de novo onset of IBS in some cases (post-infection IBS; PI-IBS) [1,6]. PI-IBS provides a paradigm for how microbiome–host interactions may play a role in IBS, in general. Serum vitamin D levels have been found to be lower in people with IBS than those without, with a high incidence of deficiency in IBS populations. resulting, perhaps, in a pro-inflammatory state given the anti-inflammatory properties of this vitamin [7]. Whilst the immunoregulatory activity of some microorganisms has prompted growing interest in the use of probiotics in IBS [8], effects are likely strain-specific [9,10].

*Bifidobacterium longum* 35624 has been shown to survive gastrointestinal transit, inhibit the growth of pathogenic organisms, prevent bacterial translocation, and exert anti-inflammatory effects [11,12]. Double-blind, placebo-controlled trials found that this strain reduced GI symptoms in adults with IBS [11,13]. Anecdotal reports from South America suggest that supplementation with this strain occurs in pediatric clinical practice. Whilst its safety has been confirmed in a pediatric population [14], data demonstrating its effectiveness among children with IBS has not been published. This real-world, open-label study conducted in Chile aimed to investigate the effects of *Bifidobacterium longum* 35624 on GI symptoms in children and adolescents with IBS and explore the relationship with baseline vitamin D status.

## 2. Materials and Methods

Ethical approval for this open-label, single-arm clinical study was obtained from the Scientific Ethics Committee of the Institute of Nutrition and Food Technology, University of Chile, and registered on clinicaltrials.gov (NCT04922476). Potential participants were identified in gastroenterology clinics by their managing physician and referred to the study physician. Children and adolescents aged 8–18 years old residing in Santiago and Coyhaique, Chile, were considered for inclusion if they met the Rome IV diagnostic criteria [5] for IBS (with or without other DGBI), reported at least two episodes of abdominal pain per week, and had moderate to severe disease severity according to the IBS severity scoring system (IBS-SSS) [15,16]. Individuals were excluded if they had systemic, organic or metabolic disease (including celiac disease), history of previous major abdominal surgery, immunosuppression, or a known allergy to any ingredients of the probiotic supplement; were pregnant or lactating; used proton-pump inhibitors, histamine type-2 receptor antagonists, fermented foods or dietary supplements containing probiotics within two weeks before the baseline visit, or antibiotics within three months before the baseline visit; or had participated in a clinical study involving an investigational product within the last two months.

Written informed consent was obtained for each potentially eligible participant from their parent or guardian. Screening assessment included documenting demographic characteristics (age, sex, height, and weight), performing a clinical history and physical examination, and obtaining blood for celiac serology (anti-endomysial and anti-transglutaminase antibodies), with clinically abnormal results leading to exclusion. Patients were also tested to define their vitamin D status (excluding those who had recently been supplemented). Participants were contacted via telephone by a research nurse one week after the baseline visit and following the review of laboratory results to confirm their participation in the study, provide instructions on taking the study product, and arrange the Week 6 and Week 12 study visits.

Participants were given the study product by the study physician (**Alflorex**^®^, produced for PrecisionBiotics Group Ltd., Cork, Ireland) containing 1 × 10^9^ colony-forming units of *Bifidobacterium longum* 35624 per capsule, to be stored at room temperature and taken once each morning with a cold drink for 12 weeks. Compliance with intake was monitored by counting the remaining capsules during the study visits at Weeks 6 and 12. This was followed by a two-week washout period during which the study product was not taken. Participants were instructed to maintain their usual dietary intake and avoid foods or other supplements containing probiotics throughout the study.

**Figure 1:**
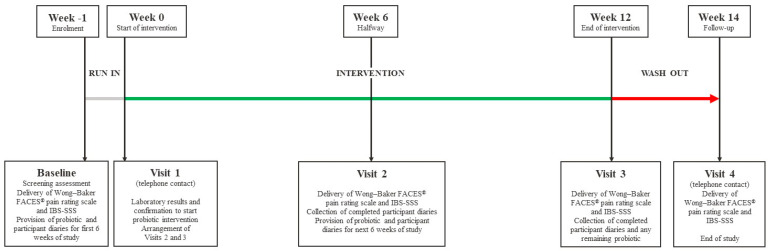
Schematic diagram of study design.

The study schematic is presented in Figure 1. Participants’ IBS symptoms were measured by the study physician at baseline (one week before the intervention period), halfway through the intervention period (Week 6 of the intervention period), at the end of the intervention period (Week 12), and at follow-up (Week 14, two weeks after the intervention period, by telephone). An adapted IBS-SSS [15,16] measured five domains: abdominal pain frequency (number of days with pain), abdominal pain severity, abdominal distension severity, bowel habit satisfaction, and impact to life, on a scale of 0–10. The Wong–Baker FACES^®^ and numeric pain rating scales [17] were used to facilitate children’s responses. Favorable changes were indicated by decreases in abdominal pain, abdominal distension, and impact to life domain scores, and by an increase in bowel habit satisfaction score. To calculate the composite IBS-SSS score, the sum of the 0–10 values for each domain was calculated and multiplied by 10. Participant diaries were provided in the local language to record parent and child perspectives on the number of IBS symptoms present during the intervention period. Questions which were suitable to be answered by both parent and child adapted from the Questionnaire on Pediatric Gastrointestinal Symptoms (QPGS) [18] were included, relating to pain/discomfort in the upper abdomen, bowel habits, other symptoms (nausea/vomiting), and those causing restriction of activities. Stool consistency (Bristol Stool Form Scale; BSFS [19]) was recorded daily during the intervention period in the participants’ diaries. The data entered during Week 1 were treated as baseline, and weekly average stool type categories were calculated for Weeks 6 and 12 as the mean of these average daily scores.

Data were analyzed primarily using an intention-to-treat (ITT) approach, with per-protocol sensitivity analysis. Descriptive statistics were calculated for the baseline characteristics, along with the proportion of participants who experienced a clinical improvement (change of ≥50 points) in composite IBS-SSS, or a numerical improvement (change of ≥1 point) in one or more IBS-SSS domains, during the intervention period. Regional differences (between study sites) in baseline vitamin D status were analyzed using an independent *t*-test, and the association between baseline vitamin D status and composite IBS-SSS with linear regression. Changes from baseline in the distribution of IBS-SSS severity categories were analyzed with a generalized linear mixed model using cumulative logits. Linear mixed-effects models were constructed to analyze the changes in composite IBS-SSS, the association between IBS-SSS treatment response and baseline vitamin D status, and the mean number of QPGS reported during the study. The distributions of average stool type categories at the same timepoints were analyzed using a generalized linear mixed model using multinominal logits. For linear mixed models, missing data were handled with restricted maximum likelihood, assuming the data were missing at random. Data are presented as mean and standard deviation (±) and percentage (%) of participants. The results of the analyses for the intention to treat population are presented. Statistical significance was set at *p* < 0.05.

## 3. Results

### 3.1. Intention to Treat Analysis

#### 3.1.1. Participant Characteristics

Between 1 August 2021 and 30 November 2022, 65 children/adolescents were included in the study. Of these, one was excluded due to a diagnosis of celiac disease, six dropped out before Week 6 of the study (*n* = 5 lost to follow-up; n = 1 unrelated adverse event: reaction to ibuprofen), and 58 completed the study and took the study product as directed (per protocol population). Baseline characteristics are presented in Table 1. In terms of vitamin D status, the majority of participants were found to be deficient (<20 ng/mL; 54.1%) or had low levels (20–30 ng/mL; 40.5%) of vitamin D. Mean vitamin D status was similar between regions (Coyhaique 17.5 ± 6.22; Santiago 20.4 ± 6.55); however, more participants from Coyhaique were deficient (77.8%) compared to Santiago (46.4%) (*p* = 0.35 not significant). All participants had IBS (mostly severe) with no significant correlation between IBS severity and vitamin D status (*p* = 0.59). Most participants had other concurrent pediatric DGBI, predominantly functional abdominal pain not otherwise specified, functional dyspepsia, and functional constipation, with a small number having non-retentive fecal incontinence. Additionally, the symptoms of bloating and nausea and the identification of a palpable bowel loop on physical examination, affected 12.5%, 1.5%, and 28.1% of participants, respectively.

#### 3.1.2. IBS Symptom Severity

**Figure 2:**
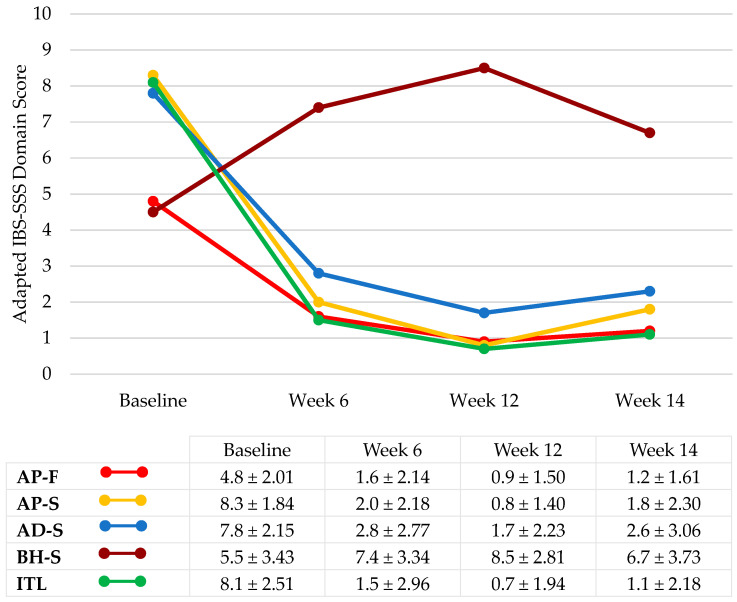
Adapted IBS-SSS domain scores during the probiotic supplementation period. IBS-SSS: irritable bowel syndrome severity scoring system; ±: standard deviation; AP-F: abdominal pain frequency; AP-S: abdominal pain severity; AD-S: abdominal distension severity; BH-S: bowel habit satisfaction; ITL: impact to life. Week 6 all *p* < 0.0001 versus baseline except BH-S (*p* < 0.0007); Week 12 all *p* < 0.0001.

During the 12-week supplementation period, there was a significant decrease in composite IBS-SSS score from baseline (334.4 ± 76.67) to Week 6 (104.3 ± 92.36, *p* < 0.0001) and Week 12 (57.6 ± 71.26, *p* < 0.0001). The mean IBS-SSS domain scores for abdominal pain frequency, abdominal pain severity, abdominal distension severity, and impact to life significantly decreased (all *p* < 0.0001 for Week 6 and Week 12 versus baseline) (Figure 2). The IBS-SSS domain score for bowel habit satisfaction significantly increased (*p* < 0.0007 for Week 6 versus baseline; *p* < 0.0001 for Week 12 versus baseline) (Figure 2). A clinically significant improvement in composite IBS-SSS was experienced by 93.1% of participants at Week 6, and 96.6% at Week 12. By Week 12, numerical improvements were experienced by all (100.0%) participants in at least two IBS-SSS domains; 98.3% in at least three domains; 91.4% in at least four domains; and 56.9% in all five domains.

During the two-week follow-up between Weeks 12 and 14, there were increases in IBS-SSS composite score (+43.21, *p* = 0.0011), abdominal pain frequency (+0.30, *p* = 0.16 not significant), abdominal pain severity (+0.97, *p* = 0.0006), abdominal distension severity (+0.91, *p* = 0.0077), and impact to life (+0.35, *p* = 0.26 not significant), and a decrease in bowel habit satisfaction (−1.82, *p* = 0.0018).

**Figure 3:**
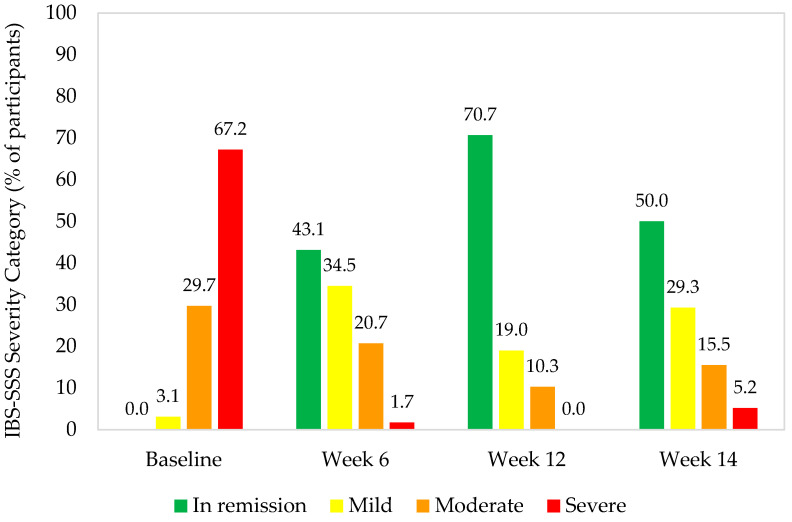
IBS-SSS severity category by timepoint (percentage of participants). IBS-SSS: irritable bowel syndrome severity scoring system.

The distribution of IBS severity categories changed from baseline to Week 12, with decreases in the proportion of participants with severe or moderate symptoms and increases in those with mild symptoms or in remission (*p* < 0.0001) (Figure 3).

#### 3.1.3. Relationship between Baseline Vitamin D Status and IBS-SSS Treatment

There was no statistically significant correlation between baseline serum vitamin D level and the change in composite IBS-SSS score from baseline to Week 6, Week 12, or Week 14 (*p* = 0.79).

#### 3.1.4. Parent and Child Perspectives of IBS Symptoms

The mean number of symptoms reported by both parent and child with the QPGS reduced from 9.6 ± 4.9 at Baseline to 6.9 ± 4.9 at Week 6 (*p* = 0.0005) and 6.9 ± 5.4 at Week 12 (*p* = 0.02).

#### 3.1.5. Stool Consistency

There was a small numerical increase in the proportion of participants who experienced ‘normal’ (BSFS stool type 3–5) weekly average stool types, which appeared to correspond to a small decrease in those experiencing constipation and, to a lesser extent, diarrhea; however, the differences between visits were not statistically significant.

### 3.2. Per Protocol Sensitivity Analysis

As the six participants who were excluded from the per-protocol analysis (9.4% of the ITT population) were all early drop-outs with insufficient outcome data beyond the baseline, the results of the per-protocol sensitivity analysis were similar to the ITT results.

## 4. Discussion

This real-world, open-label study conducted in Chile showed significant reductions in the severity and prevalence of IBS symptoms in children and adolescents with IBS and concurrent DGBI during 12 weeks of supplementation with *B. longum* 35624. The findings provide evidence to support the effectiveness of this immunoregulatory probiotic strain in a vitamin D-deficient population.

This is the first study to report on the effectiveness of this specific probiotic strain in a pediatric IBS population. Few published studies from Latin America have investigated the action of other probiotic strains in children with IBS, reporting contradictory findings regarding their effects on GI symptom severity [20,21]. The clinical features of the pediatric population in this region warrant specific investigation, with a higher prevalence of childhood constipation in South America than in other countries [22], accounting for one-third of pediatric gastroenterology consultations in Chile [23]. Disease overlap between IBS and functional defecation disorders is common and has been associated with increased symptom severity and decreased quality of life [24]. In the present study, the majority of participants had other DGBI including two-thirds with functional constipation, in addition to moderate or severe IBS at baseline, yet the probiotic proved to be highly effective.

During the 12-week intervention with *B. longum* 35624, there were significant improvements in all IBS-SSS domain scores, including abdominal pain severity and frequency, abdominal distension severity, and bowel habit satisfaction, with a high proportion of participants experiencing improvements across multiple domains. This culminated in a clinically significant decrease in IBS-SSS composite score, and a distributional shift towards less severe IBS-SSS severity categories, reflecting the findings of published literature from adult IBS populations [11,13,25]. Although the IBS-SSS bowel habit satisfaction score improved, statistically significant changes in stool consistency were not observed. Similar findings from randomized, placebo-controlled trials of *B. longum* 35624 in adults with IBS have suggested that the effectiveness of this *Bifidobacterium* strain may be applicable to all patients with IBS, irrespective of stool pattern [11,13].

The prevalence of a broader range of GI symptoms reported by both parents and children/adolescents also improved during the study, with significantly fewer QPGS affecting participants after the probiotic intervention compared to baseline. This offers an important insight, as research has found that patient and doctor perspectives of IBS severity and its impact to life differ, which may compromise quality of life [26]. In children, IBS is known to impair physical, social, emotional, and school functioning [27] and quality of life [28], indicating the need for targeted approaches to address these aspects of well-being. In the present study, improvements in IBS symptoms were accompanied by a significant decrease in the impact of IBS to daily life, which is consistent with published real-world data demonstrating reductions in symptom severity and increased QoL in adults with IBS following a four-week intake of *B. longum* 35624 [25].

The observed improvements in IBS-SSS scores were evident from week six and peaked at week 12, reflecting recent real-world data trends from an adult IBS population in Russia, which showed cumulative improvements with *B. longum* 35624 over a 12 week period, and persistence of these effects for up to one month after probiotic intervention [29]. Conversely, randomized placebo-controlled trials have demonstrated that the positive effects of this strain on IBS symptoms were not maintained long after the cessation of intake. Similar results were observed in the present study, with statistically significant increases in IBS-SSS scores during the two-week washout period. This may suggest that this microorganism may not permanently colonize the bowel, with continuous intake required for long-term effects [11,13].

Another interesting finding of this study was the high prevalence of vitamin D deficiency in this cohort. A high prevalence of vitamin D deficiency has been reported among children in Chile, which has been attributed to the use of sunscreen, indoor activity, diet, and obesity in this region [30]. Although previous studies have shown a higher incidence of deficiency and lower serum levels of vitamin D in IBS populations, the relationship between vitamin D status and symptom severity is unclear [7]. The present study detected no correlation between baseline vitamin D status and the severity of IBS symptoms. Despite its immunoregulatory and anti-inflammatory properties and its impact on the gut microbiome, a recent meta-analysis reported significant improvements in quality of life but not symptom severity with vitamin D supplementation in people with IBS [7].

Additionally, no statistically significant correlation between baseline vitamin D status and probiotic response was detected in the present study. *B. longum* 35624 has been shown to bypass the inflammatory nuclear factor-kappa B pathway which typifies immune response to a pathogen, instead engaging with dendritic cells to initiate an anti-inflammatory, regulatory T-cell pathway [31]. The clinical significance of this has been demonstrated by reductions in C-reactive protein and pro-inflammatory cytokines tumor necrosis factor (TNF)-α and interleukin (IL)-6 in adults with inflammatory conditions [32], and restoration of a favorable IL-10:IL-12 ratio which accompanied symptom improvements in adults with IBS [11]. In this study of children and adolescents with IBS, significant improvements in GI symptoms were observed in those with adequate and low serum vitamin D levels at baseline, confirming the effectiveness of *B. longum* 35624 in a vitamin D deficient population. Further research is required to determine whether other probiotic strains are also effective in individuals with low vitamin D levels, and the extent to which vitamin D status influences probiotic activity in the host.

The absence of a comparator group was a key limitation of this open-label study. A notable placebo effect has been reported in IBS studies [33], with an average decrease in composite IBS-SSS score of 30 points with placebo having been reported [34]. However, the size of the reduction in composite IBS-SSS score in the current study, exceeding 270 points, the high proportion of participants who experienced a 50-point or more reduction, which has been validated as a reliable indicator of clinical improvement [15], and the consistency of these results with those of double-blind, placebo-controlled trials of *B. longum* 35624 in adult IBS populations [11,13] are suggestive of a true probiotic effect. Although there was a nine percent drop-out rate and missing data partly due to the impact of the 2019 coronavirus pandemic that inhibited the analysis of exploratory outcomes, the use of ITT analysis resulted in a conservative estimation of probiotic effect on the IBS symptom scores. As potential participants were identified and referred by clinicians from study sites in Santiago and Coyhaique, the study population can be considered representative of the local pediatric gastroenterology population. A randomized placebo-controlled trial with a larger sample size may be required to confirm the generalizability of the findings.

## 5. Conclusions

The findings of this real-world study contributed to the evidence base for the use of *B. longum* 35624 in the management of IBS symptoms, confirming its effectiveness in a pediatric population even in the presence of vitamin D deficiency. Future well-designed studies in a larger cohort are warranted to confirm these findings.

## Figures and Tables

**Table 1:** Baseline characteristics of participants.

Characteristic	n = 64
**Demographics**	
Female, n (%)	42 (65.6)
Age (years), mean ± SD	12.3 ± 2.9
Resident of Santiago, n (%)	47 (73.4)
Resident of Coyhaique, n (%)	17 (26.6)
**Anthropometrics, mean ± SD**	
Weight (kg)	45.7 ± 15.3
Height (cm)	148.1 ± 15.1
BMI (kg/m^2^)	20.3 ± 4.0
**Biochemistry, mean ± SD**	
Serum vitamin D *, ng/mL (sufficiency ≥ 30 ng/mL)	19.7 ± 6.50
**Concurrent pediatric DGBI (Rome IV criteria [5]), n (%)**	
H2. Functional abdominal pain disorders	64 (100.0)
H2a. Functional dyspepsia	45 (70.3)
H2b. Irritable bowel syndrome (IBS)	64 (100.0)
H2c. Abdominal migraine	0 (0.0)
H2d. Functional abdominal pain not otherwise specified	60 (93.8)
H3. Functional defecation disorders	60 (93.8)
H3a. Functional constipation	43 (67.2)
H3b. Non-retentive fecal incontinence	9 (14.1)
Functional defecation disorder not otherwise specified	8 (12.5)
**IBS severity**	
Participants in IBS remission (IBS-SSS < 75), n (%)	0 (0.0)
Participants with mild IBS (IBS-SSS 75–175), n (%)	2 (3.1)
Participants with moderate IBS (IBS-SSS 176–300), n (%)	19 (29.7)
Participants with severe IBS (IBS-SSS > 300), n (%)	43 (67.2)

±SD: standard deviation; BMI: body mass index; DGBI: disorders of gut–brain interaction; IBS-SSS: irritable bowel syndrome severity scoring system. * n = 37.

## Data Availability

The original contributions presented in the study are included in the article, further inquiries can be directed to the corresponding author.
